# Comparative analysis of laser and ultrasonic irrigation techniques for smear layer removal in endodontics

**DOI:** 10.1007/s10103-025-04766-4

**Published:** 2025-12-13

**Authors:** Muhammad Mahmoud Abaza, Tarek Abdel Hamid Harhash, Ahmed Abbas Zaky

**Affiliations:** Medical Laser Applications Department, National Institute of Enhanced Laser Sciences, Cairo University, Giza, Egypt

**Keywords:** Er, Cr:YSGG laser, Diode laser, Laser-activated irrigation, Radial firing tip, Smear layer removal

## Abstract

**Objective:**

This in vitro study compared the efficacy of Er, Cr: YSGG laser (2780 nm), diode laser (976 nm), passive ultrasonic irrigation (PUI), and conventional syringe-needle (CSN) irrigation in smear layer removal, evaluating irrigant chemistry and root canal level.

**Methods:**

160 single-rooted premolars were instrumented and divided into four irrigation groups (*n* = 40/group): Er, Cr: YSGG (25 mJ, 50 Hz, radial-firing tip), diode laser (Pulsed 50%, 1.5 W), PUI, and CSN (side-vented needle). Groups were subdivided by irrigant (NaOCl + EDTA, EDTA, NaOCl, saline; *n* = 10/subgroup). Activation involved four 15-second cycles. Smear layer was scored (1–5) via SEM by blinded evaluators. Data were analyzed with Kruskal-Wallis and Mann-Whitney tests (*p* ≤ 0.05).

**Results:**

Er, Cr: YSGG achieved the lowest scores (best cleaning) across all thirds, outperforming diode laser, PUI, and CSN. NaOCl + EDTA was the most effective irrigant (*p* < 0.05). Remarkably, saline with Er, Cr: YSGG surpassed NaOCl alone. Apical thirds showed consistently poorer removal.

**Conclusion:**

Er, Cr: YSGG laser activation with NaOCl + EDTA is the most effective protocol for smear layer removal. The powerful mechanical effects of Er, Cr: YSGG dominated when using saline. Diode laser activation also surpassed PUI.

## Introduction

 The success of root canal therapy depends on the thorough elimination of microbes, necrotic pulp tissue, and inorganic debris from the root canal system [1, 2]. Nevertheless, the complex anatomy of root canals—featuring isthmuses, lateral canals, and apical deltas—poses a major challenge to achieving complete debridement with conventional techniques [2, 3].

Furthermore, a smear layer, a 1–5 μm thick deposit of organic and inorganic debris produced during instrumentation, consistently obstructs the dentinal walls and tubules of the main canals [2]. This layer can act as a reservoir for pathogens, impede the penetration of irrigants and sealing materials, and compromise the three-dimensional seal of root canal fillings [2]. Consequently, the effective removal of the smear layer is a critical prerequisite for optimal decontamination and the adhesion of obturation materials [2]. Presently, the most efficient method for eliminating this smear layer is the root canal irrigation process [2]. However, the irrigation protocols employed for its removal also chemically and structurally alter the underlying dentin substrate, which can significantly impact the adhesion of obturation materials and the long-term mechanical integrity of the root [4–6]. For instance, recent scoping reviews highlight that irrigants like NaOCl and EDTA can alter the collagen network and mineral content of dentin, thereby influencing its bonding capacity and biomechanical properties [4–6]. Consequently, an ideal irrigation strategy must balance maximal debridement with the preservation of dentin biocompatibility and strength.

Despite its common use, the conventional syringe-needle irrigation (CSN) technique with open-ended needles is restricted in its capacity to reach complex root canal spaces due to vapor lock effects and poor fluid dynamics [7]. As a result, side-vented irrigation needles were developed for their capacity to enable deeper and more controlled irrigant delivery, thereby enhancing the effectiveness of the CSN irrigation method [8]. Unlike open-ended endodontic needles, which dispense irrigant solely from the tip, side-vented needles distribute fluid laterally, reducing apical pressure and improving debridement in the middle and coronal thirds [8]. However, their ability to eliminate the smear layer—particularly in the apical third—remains limited and inferior to that of active endodontic irrigation methods [9]. To address these limitations, advanced irrigation activation techniques such as manual dynamic activation (MDA), sonic irrigation (SI), passive ultrasonic irrigation (PUI), and laser activation have been investigated [3, 7–9].

PUI is a technique where a non-cutting ultrasonic tip is oscillated passively within the pre-shaped canal to activate the irrigant fluid [10]. The strong cleaning ability of ultrasonic energy, achieved through acoustic streaming and cavitation, supports the use of PUI to enhance irrigant penetration and debris removal [9]. Although the clinical significance of cavitation in PUI remains debated [9], multiple in-vitro studies have confirmed PUI’s effectiveness in removing smear layers, especially in the apical third where CSN often proves inadequate [9]. However, these studies also noted that improper use of PUI may lead to tip fracture [8] or dentinal microcracks [11].

Recently, optical activation has emerged as a viable alternative to sonic and ultrasonic stimulation for root canal irrigation. Dental lasers can be used to disinfect both dry and wet root canals [12, 13]. However, directly irradiating the root canal dentin with a laser beam can result in unwanted ablation and thermal damage [12, 13]. Instead, exposing the irrigant solution itself to laser energy improves its fluid dynamics, penetration, disinfection potency, and cleansing effect. This leads to deeper sealer penetration and enhances circumferential sealing and adaptation to the dentin walls [2, 14, 15].

Conventional laser-activated irrigation (LAI) first utilized mid-infrared (MIR) lasers before shifting to the use of near-infrared (NIR) lasers [12, 13, 16]. These lasers are delivered through optical fibers and tips that can reach nearly the full working length of the canal, enabling effective agitation of the irrigant in the apical region [13, 16, 17]. Although NIR wavelengths—such as those from 810, 940, and 980-nm diode lasers—are mainly absorbed by pigmented chromophores instead of water, multiple in vitro studies confirm that the energy from diode laser is sufficiently absorbed by irrigants to induce effective fluid agitation [13, 18–20]. Furthermore, other studies have found that applying a continuous-wave and pulsed infrared laser beam during LAI can increase the temperature of the irrigant, thereby enhancing its kinetic movement within the canal. The required helical movement of the laser tip during the delivery of IR laser energy can also positively influence fluid dynamics. In summary, the primary interaction mechanism between NIR lasers (both CW and pulsed) and root canal irrigants is photothermal [12, 19, 21].

In contrast to NIR lasers, the primary mechanism for MIR laser-irrigant interaction is photomechanical, though it is initiated by the thermal effect of laser photons. The strong absorption of MIR radiation—such as that from Erbium family lasers—by water molecules in the irrigant, along with the conversion of light energy into heat, causes rapid and transient superheating. This results in the formation of an expanding vapor bubble, which is followed by secondary photomechanical effects such as cavitation, acoustic streaming, and shock wave generation [16, 17, 22–26]. Conventional LAI using microsecond-pulsed Er, Cr: YSGG (2.78 μm) and Er: YAG (2.94 μm) lasers has traditionally employed end-firing tips [16, 17, 22]. However, the design of these tips limits uniform irradiation of the entire root canal surface, resulting in incomplete disinfection and debris removal. To overcome this drawback, radial-firing tips (RFTs) were developed to enable divergent laser beam delivery, providing broader coverage of the canal walls. This advancement enhances fluid agitation, improves smear layer removal, and increases the overall effectiveness of activated irrigation [25, 27, 28].

Therefore, this study was conducted to compare four root canal irrigation techniques for smear layer removal: a pulsed MIR Er, Cr: YSGG laser (2.78 μm) with RFT, a continuous-wave NIR diode laser (976 nm), PUI, and CSN using a side-vented needle. The performance of these techniques was assessed using the following irrigants: 5.25% NaOCl + 17% EDTA, 17% EDTA alone, 5.25% NaOCl alone, and saline. The primary null hypothesis of this study was there will be no statistically significant difference in smear layer removal efficacy between Er, Cr: YSGG laser-activated irrigation, diode laser, PUI, and conventional needle irrigation, regardless of the irrigant solution used.

## Materials & methods

### Sample selection, grouping, and preparation

The study protocol was approved by the institutional review board (approval no. NILES-EC-CU 23/9/21 [in]). A total of 160 teeth were selected and divided into four experimental groups. The sample size was calculated using G*Power software (v3.1.9.7, Heinrich-Heine Universität, Germany). Based on previous comparable in vitro studies on smear layer removal that reported an effect size of 0.4 [20, 29–31], it was selected for this calculation, with an alpha error probability of 0.05, resulting in an achieved power greater than 0.98.

The research utilized freshly extracted mandibular first premolars obtained from patients aged 18–25 years who were undergoing orthodontic treatment [30]. The inclusion criteria specified that teeth must be intact, single-rooted, possess an oval-shaped single canal, have a mature apex, and exhibit minimal root curvature (< 20°) [14, 15, 20, 30, 32]. After extraction, external debris was cleaned from the teeth using an ultrasonic scaler and tap water [20, 32]. The teeth were then stored in normal saline based on several previous studies [10, 11, 20, 30, 33] at a temperature of + 4 °C [20, 30] and used within five days [20, 30]. It is acknowledged that saline prolonged storage may lead to dentin demineralization over time, and future studies may benefit from using isotonic solutions like Hank’s Balanced Salt Solution (HBSS) to better preserve the dentin’s organic and inorganic balance.

Digital radiographs taken from buccolingual and mesiodistal views confirmed the standardized root canal cross-section and the absence of radicular morphological anomalies, calcifications, cracks, fractures, or resorptive lesions [14, 15, 20, 32]. A low-speed diamond saw (Isomet 4000, Buehler Ltd., USA) was employed to decapitate the teeth horizontally under water cooling, standardizing the root length to 17 mm [20]. Canal patency was confirmed using a #10 K-file (Dentsply Sirona, Switzerland) [14, 15, 20, 32]. The working length was determined by subtracting 0.5 mm from the length where the file tip was first visible at the apical foramen [14, 15, 20, 32]. To prevent irrigant extrusion and simulate clinical conditions, the root apices were sealed externally with sticky wax [20, 32].

Canal preparation was performed using ProTaper Next^®^ rotary files (Dentsply Maillefer, USA) in conjunction with an endodontic motor (X-Smart, Dentsply Maillefer, USA) set at 300 rpm and 2 N/cm torque [15, 32]. Following each instrumentation cycle, the canals were rinsed with 2 mL of distilled water [14, 15] for 1 min [34] using a conventional syringe with a 30-gauge side-vented needle to standardize the initial smear layer formation [35].

### Final irrigation protocols

A computer-generated random number sequence (www.random.org) was used to allocate the 160 specimens into the four main experimental groups (*n* = 40 per group) according to the irrigation activation method used: Er, Cr: YSGG laser (Group I), diode laser (Group II), PUI (Group III), and CSN (Group IV) (see Table 1).

**Table 1:** Experimental groups and subgroups

Group	Activation Method	Subgroups (Irrigants)
I	Er, Cr: YSGG (2780 nm)	NaOCl + EDTA, EDTA, NaOCl, Saline
II	Diode Laser (976 nm)	NaOCl + EDTA, EDTA, NaOCl, Saline
III	PUI	NaOCl + EDTA, EDTA, NaOCl, Saline
IV	CSN (side-vented needle)	NaOCl + EDTA, EDTA, NaOCl, Saline

- *Group I (Er*,* Cr: YSGG laser activation)*: The irrigant was activated using a 2.78-µm Er, Cr: YSGG laser (iPlus, Waterlase, Biolase Technology, Irvine, CA, USA) with a RFT measuring 415 μm in diameter and 21 mm in length (Waterlase laser tip RF3-21, Biolase Technology, Irvine, CA, USA). The laser parameters were set to a pulse energy of 25 mJ [14, 18, 32], a pulse width of 60 µs [14, 18, 32, 36], and a frequency of 50 Hz [14, 18, 32].
- *Group II (Diode laser activation)*: Activation was performed using a 976-nm AlGaAs diode laser (LX 16 Plus Dental Diode Laser, Guilin Woodpecker Medical Instrument Co. Ltd., China) with a 400-µm diameter fiber optic tip (MF4-9, Guilin Woodpecker Medical Instrument Co. Ltd., China). The laser was operated in pulsed mode (50% duty cycle) at a power output of 1.5 W [20].

Prior to activation in both laser groups (I and II), the tips were placed 2 mm short of the apex [14, 15, 20, 32]. A rubber stopper was used on the laser fiber to ensure precise positioning. During the procedure, the tips were moved in a helical, apico-coronal motion at a speed of 1 mm per second [14, 15, 20, 32, 37].

- *Group III (PUI)*: The irrigant was activated using a #20/02 ultrasonic tip (Ultra X Silver, Eighteeth, Changzhou, China) connected to a wireless ultrasonic device (Ultra X, Eighteeth, Changzhou, China) operating at a frequency of 45 kHz. This flexible tip was positioned 1 mm short of the apex during activation [38].
- *Group IV (CSN)*: The irrigant was delivered passively using a 30-gauge side-vented needle (Monoject, Sherwood Medical, Switzerland) placed 1 mm short of the working length, with no activation performed [15].

Each main group was subdivided into four subgroups (*n* = 10 per subgroup) based on the irrigant solution: 5.25% NaOCl + 17% EDTA (subgroup I), 17% EDTA alone (subgroup II), 5.25% NaOCl alone (subgroup III), and normal saline (subgroup IV).

Subgroups employing EDTA alone, NaOCl alone, and saline were included as experimental controls to delineate the individual chemomechanical effects of each irrigant and to evaluate the pure mechanical agitation efficacy of each activation system.

For the activated irrigation groups (I-III), the process consisted of four 15-second activation cycles with 5-second intervals, resulting in a total agitation time of 60 s. The activation was synchronized with irrigant application, starting and ending simultaneously. The non-activated group (IV) received irrigation over four corresponding 15-second cycles for an equivalent total duration.

All subgroups, except for subgroup I, received a total of 6 mL of a single irrigant solution, delivered in 4 cycles of 1.5 mL each. In contrast, the root canals in subgroup I underwent sequential irrigation with a total volume of 6 mL: 3 mL of NaOCl delivered over 2 cycles (1.5 mL per cycle), followed by a rinse with 2 mL of distilled water, and then 3 mL of EDTA delivered over another 2 cycles (1.5 mL per cycle).

### SEM analysis

Following irrigation, the canals were flushed with 2 mL of distilled water [32] and subsequently dried using #X4 absorbent paper points (ProTaper^®^ Next, Dentsply Sirona, Ballaigues, Switzerland). A temporary gutta-percha cone of size #X4 (ProTaper^®^ Next, Dentsply Sirona, Ballaigues, Switzerland) was placed in the canal to serve as a guide during groove preparation, prevent perforation, and avoid contamination from debris during root splitting [14, 15, 32]. Grooves were then prepared along the entire buccal and lingual surfaces of each root, parallel to its long axis, without entering the root canal space. The two connected root halves were carefully separated using a stainless-steel chisel [20, 32]. One half of the root was chosen for examination under an environmental scanning electron microscope (ESEM) (FEI Quanta 250 FEG, Berlin, Germany) at 2000× magnification and an electron accelerating voltage of 20 kV [14, 15, 32].

**Fig. 1:**
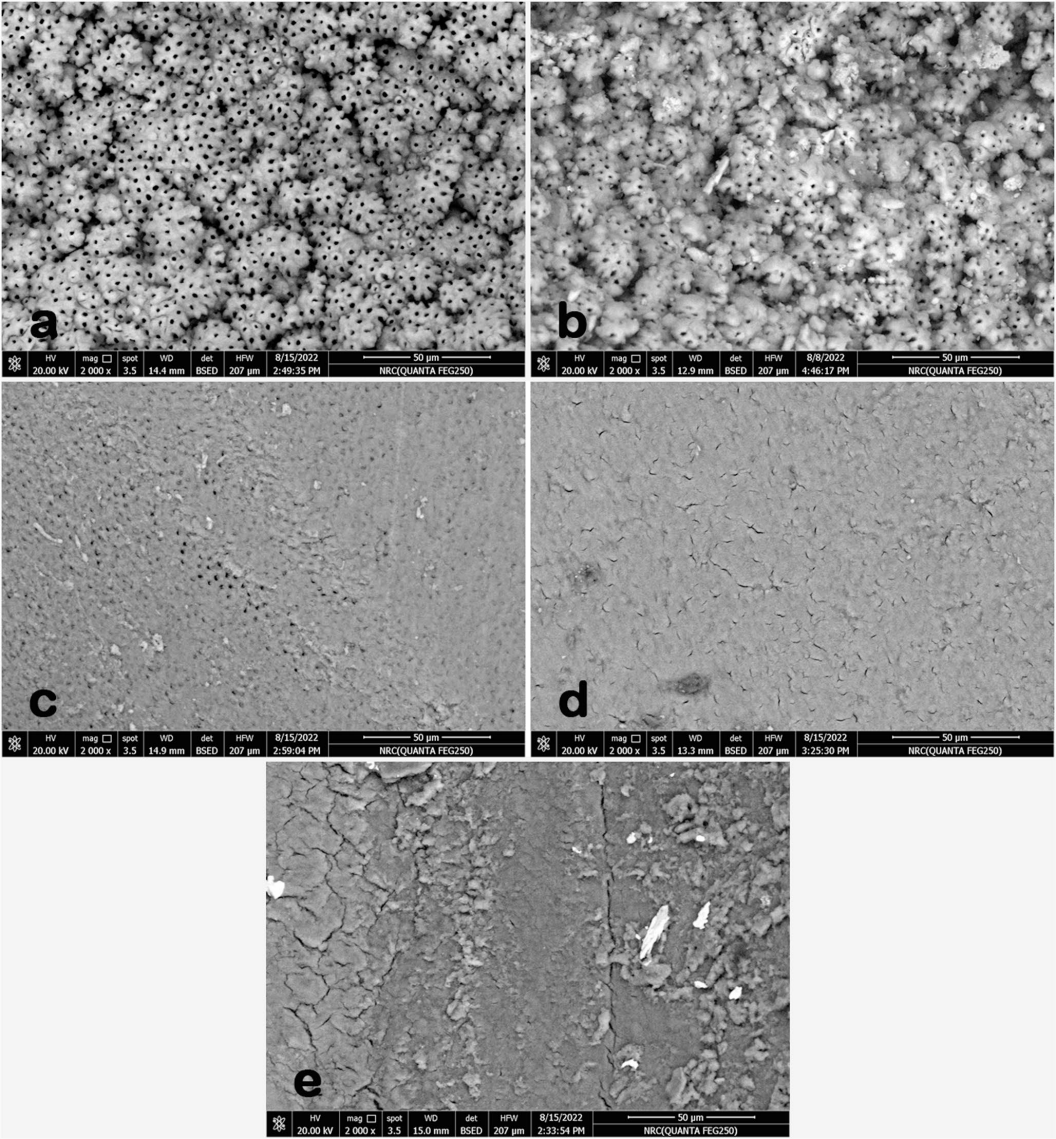
Representative SEM images categorized by a 5-point scoring scale: (**a**) Score 1: Full dentinal tubules visibility without smear layer, (**b**) Score 2: Partial tubule visibility with minor smear layer. (**c**) Score 3: Most dentinal tubules are obscured with moderate smear layer. (**d**) Score 4: All dentinal tubules are obscured with homogeneous smear layer covering the entire canal surface. (**e**) Score 5: All dentinal tubules are obscured with heterogeneous, heavy, irregular smear layer covering the entire canal surface [16]

The removal of the smear layer from the coronal, middle, and apical thirds was evaluated blindly by two independent, trained examiners [14, 15, 32] using the five-point scoring system established by Hülsmann et al. [39]. The scoring criteria were defined as follows (see Fig. 1):

- Score 1: Complete visibility of dentinal tubules with no smear layer.
- Score 2: Partial visibility of dentinal tubules with minimal smear layer.
- Score 3: Majority of dentinal tubules obscured by moderate smear layer.
- Score 4: All dentinal tubules obscured by a homogeneous smear layer covering the entire canal surface.
- Score 5: All dentinal tubules obscured by a heterogeneous, thick, irregular smear layer covering the entire canal surface.

### Statistical analysis

The data obtained from ESEM images were analyzed using SPSS (Statistical Package for the Social Sciences, version 25.0.0, IBM, Armonk, NY, USA). Inter-examiner and intra-examiner reliability were assessed and confirmed using Cohen’s kappa coefficient. Normality testing performed with the Kolmogorov-Smirnov and Shapiro-Wilk tests indicated a non-normal distribution of the data. Consequently, nonparametric statistical tests were selected for analysis. The Kruskal-Wallis test was employed for overall intergroup comparisons (categorized by root canal irrigant type and root third), while the Mann-Whitney U test was used for pairwise comparisons. A p-value of ≤ 0.05 was considered statistically significant.

## Results

### Comparison of irrigation techniques

**Fig. 2:**
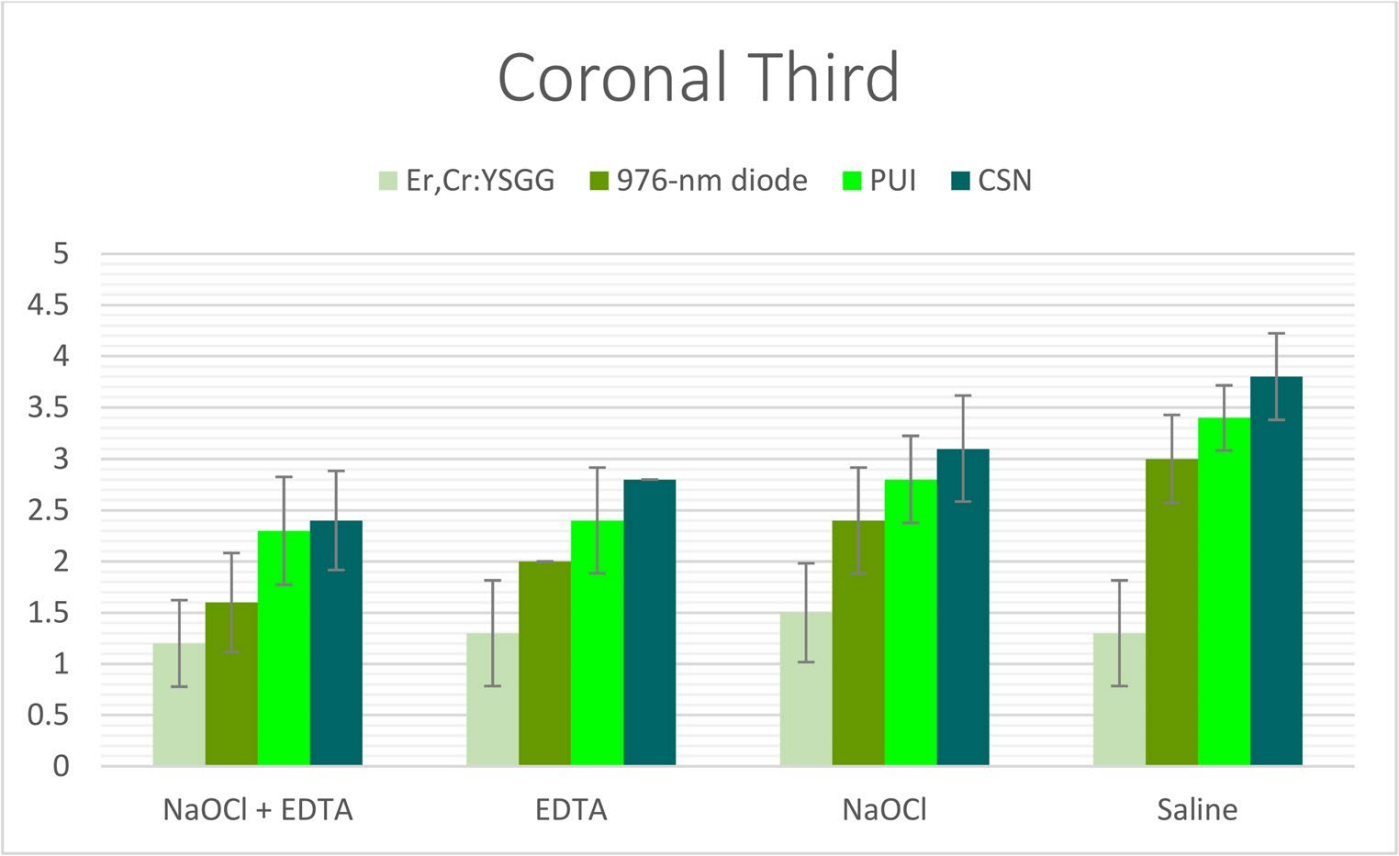
The mean of smear layer scores of all groups recorded at coronal third

**Fig. 3:**
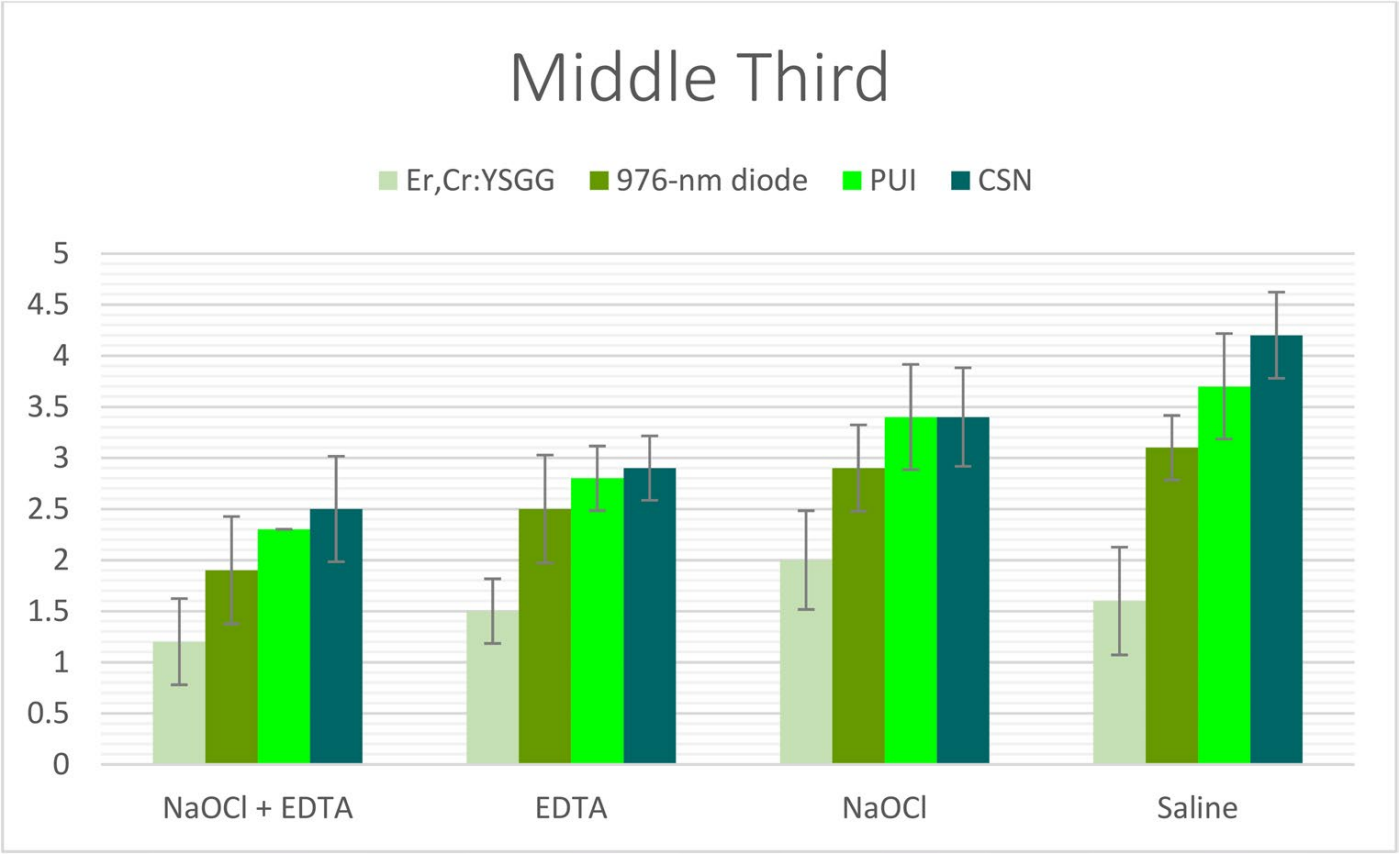
The mean of smear layer scores of all groups recorded at middle root third

**Fig. 4:**
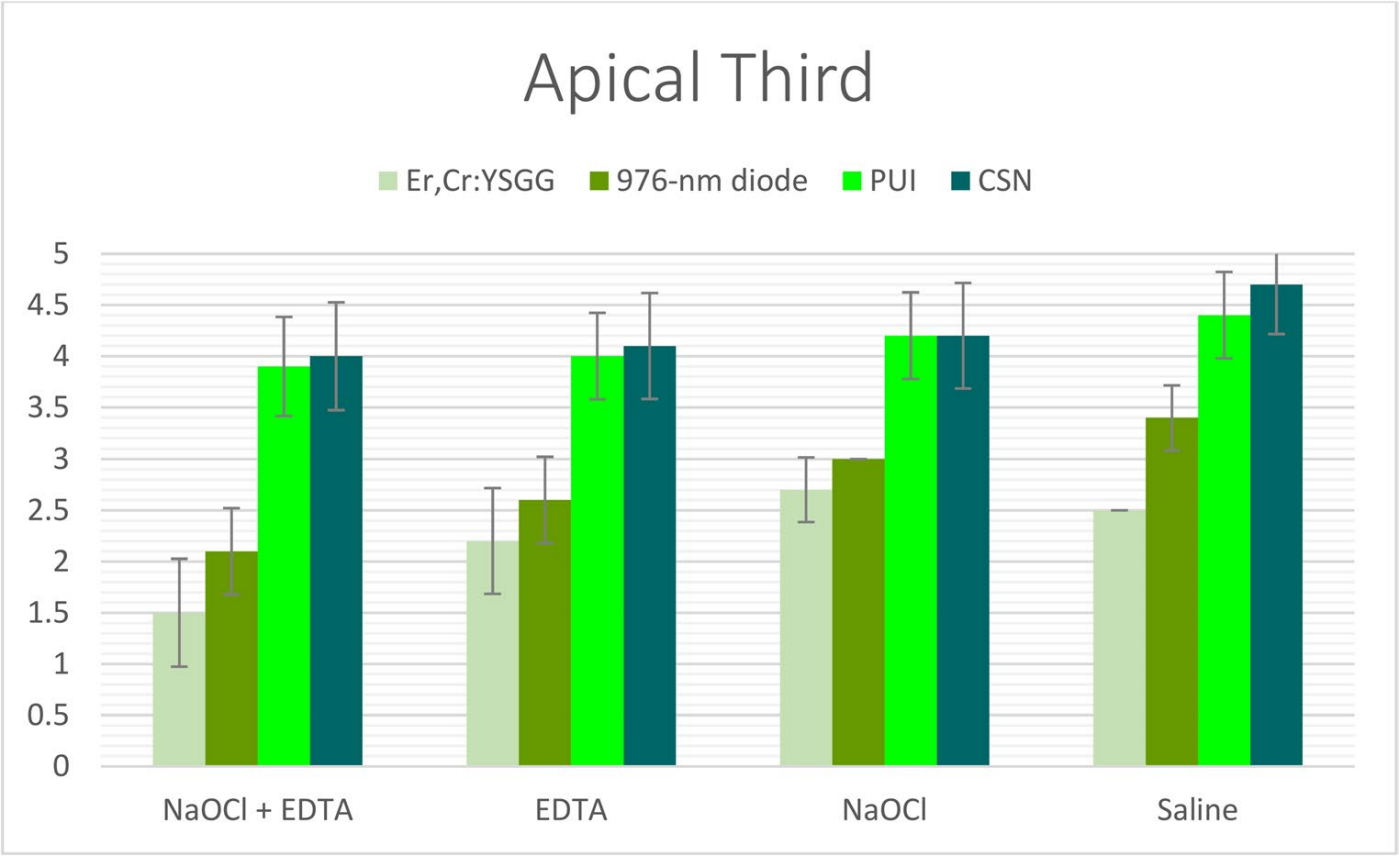
The mean of smear layer scores of all groups recorded at apical root third

Statistically significant differences in smear layer removal efficacy were observed among the four experimental groups (*p* < 0.001), irrespective of the irrigant solution or root canal level evaluated. The Er, Cr: YSGG laser-activated irrigation technique (Group I) consistently demonstrated superior performance over all other methods, achieving the lowest smear layer scores across all root canal thirds, regardless of the irrigant used (Tables 2, 3 and 4; Figs. 2, 3 and 4). The diode laser-activated irrigation method (Group II) showed significantly better results than both PUI (Group III) and CSN (Group IV). Furthermore, PUI (Group III) exhibited significantly greater smear layer removal efficacy than CSN (Group IV) specifically when NaOCl + EDTA was applied in the coronal third and when saline was used in the middle third (Tables 2, 3 and 4; Figs. 2, 3 and 4).

**Table 2:** Mean, standard deviation (SD), and the statistical comparisons of smear layer scores in coronal third between the examined groups & subgroups

	NaOCl + EDTA	EDTA (17%)	NaOCl (5.25%)	Saline	Kruskal-Wallis H	*P*-value
Er, Cr: YSGG	1.2 ± 0.422 A, a	1.3 ± 0.483 A, a	1.5 ± 0.527 A, a	1.3 ± 0.483 A, a	2.1111	0.550
976-nm diode	1.6 ± 0.516 B, a	2 ± 0 B, b	2.4 ± 0.516 B, c	3 ± 0 B, d	28.044	< 0.001*
PUI	2.3 ± 0.483 C, a	2.4 ± 0.516 C, ab	2.8 ± 0.422 C, b	3.4 ± 0.516 C, c	17.549	0.001*
CSN	2.4 ± 0.516 D, a	2.8 ± 0.422 C, ab	3.1 ± 0.316 C, b	3.8 ± 0.422 C, c	24.190	< 0.001*
*Kruskal-Wallis H*	21.486	25.666	25.898	31.050		
*P-value*	< 0.001*	< 0.001*	< 0.001*	< 0.001*		

- (*) Values had statistically significant difference at (*P* < 0.05).

*- *Different lowercase letters (a, b, c, d, e) indicate significant differences within each row (*P* < 0.05).

- Different uppercase letters (A, B, C) indicate significant differences within each column (*P* < 0.05).

**Table 3:** Mean, standard deviation (SD), and the statistical comparisons of smear layer scores in middle third between the examined groups & subgroups

	NaOCl + EDTA	EDTA (17%)	NaOCl (5.25%)	Saline	Kruskal-Wallis H	*P*-value
Er, Cr: YSGG	1.2 ± 0.422 A, a	1.5 ± 0.527 A, a	2 ± 0 A, b	1.6 ± 0.516 A, a	13.066	0.004*
976-nm diode	1.9 ± 0.316 B, a	2.5 ± 0.527 B, b	2.9 ± 0.316 B, bc	3.1 ± 0.316 B, c	25.337	< 0.001*
PUI	2.3 ± 0.483 C, a	2.8 ± 0.422 B, b	3.4 ± 0.516 C, c	3.7 ± 0.483 C, c	22.870	< 0.001*
CSN	2.5 ± 0.527 C, a	2.9 ± 0.316 B, a	3.4 ± 0.516 C, b	4.2 ± 0.422 D, c	28.026	< 0.001*
*Kruskal-Wallis H*	22.857	23.100	28.033	32.713		
*P-value*	< 0.001*	< 0.001*	< 0.001*	< 0.001*		

- (*) Values had statistically significant difference at (*P* < 0.05).

*- *Different lowercase letters (a, b, c, d, e) indicate significant differences within each row (*P* < 0.05).

- Different uppercase letters (A, B, C) indicate significant differences within each column (*P* < 0.05).

**Table 4:** Mean, standard deviation (SD), and the statistical comparisons of smear layer scores in apical third between the examined groups & subgroups

	NaOCl + EDTA	EDTA	NaOCl (5.25%)	Saline	Kruskal-Wallis H	*P*-value
Er, Cr: YSGG	1.5 ± 0.527 A, a	2.2 ± 0.422 A, a	2.7 ± 0.483 A, a	2.5 ± 0.527 A, b	18.068	< 0.001*
976-nm diode	2.4 ± 0.516 B, a	2.8 ± 0.422 B, a	3.2 ± 0.422 B, b	3.6 ± 0.516 B, c	19.500	< 0.001*
PUI	3.9 ± 0.316 C, a	4 ± 0 C, a	4.2 ± 0.422 C, ab	4.4 ± 0.516 C, b	9.066	0.028*
CSN	4 ± 0 C, a	4.1 ± 0.316 C, ab	4.2 ± 0.422 C, b	4.7 ± 0.483 C, c	15.080	0.002*
*Kruskal-Wallis H*	35.267	35.520	30.505	29.161		
*P-value*	< 0.001*	< 0.001*	< 0.001*	< 0.001*		

- (*) Values had statistically significant difference at (*P* < 0.05).

- Different lowercase letters (a, b, c, d, e) indicate significant differences within each row (*P* < 0.05)*.*

- Different uppercase letters (A, B, C) indicate significant differences within each column (*P* < 0.05).

### Comparison of root Canal solutions

**Fig. 5:**
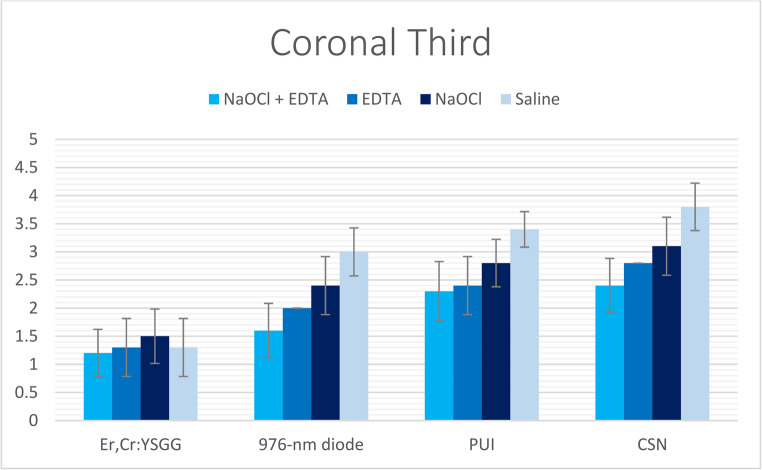
The mean of smear layer scores of subgroups with each group recorded at coronal root third

**Fig. 6:**
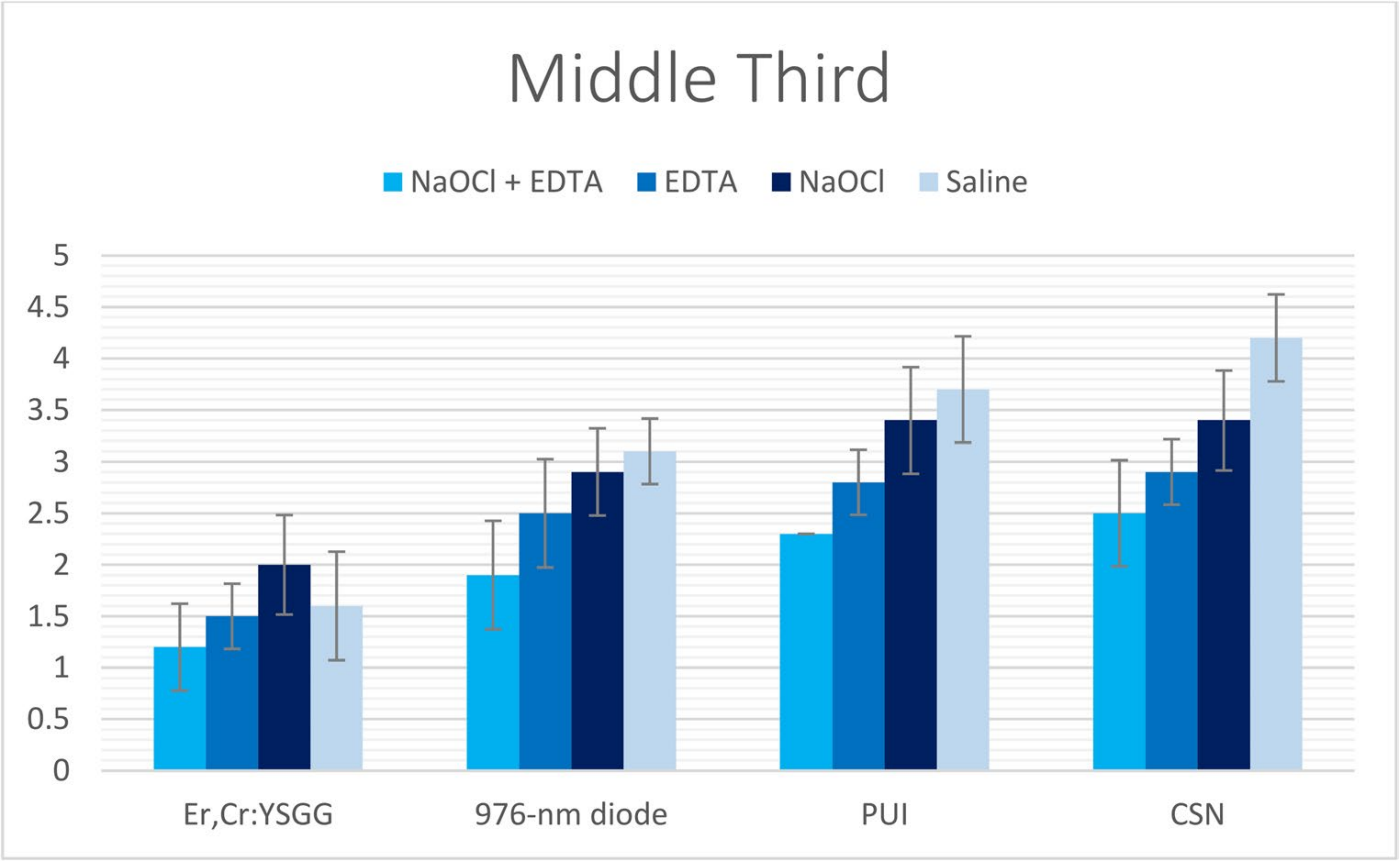
The mean of smear layer scores of subgroups with each group recorded at middle root third

**Fig. 7:**
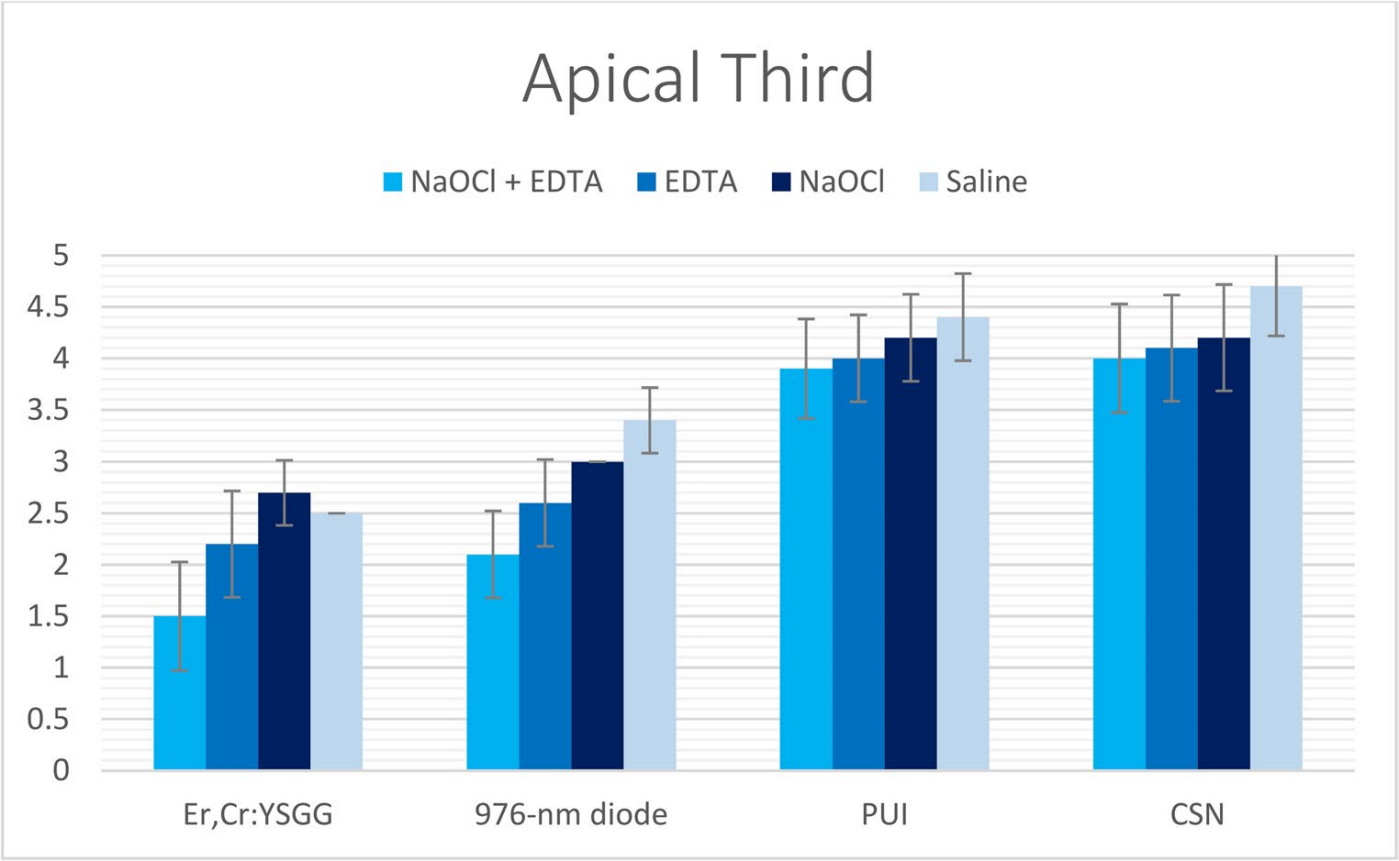
The mean of smear layer scores of subgroups with each group recorded at apical root third

When irrigants were compared under identical irrigation protocols, the combination of NaOCl + EDTA (Subgroup I) demonstrated the highest level of smear layer removal, exceeding the performance of all other irrigants across every root segment. This was followed by EDTA alone (Subgroup II). Conversely, NaOCl used alone (Subgroup III) consistently showed inferior smear layer removal compared to saline (Subgroup IV) in all root segments under the same irrigation protocol—with the sole exception of when the Er, Cr: YSGG laser-activated technique (Group I) was employed. Notably, within Group I, saline (Subgroup IV) exhibited greater efficacy than NaOCl alone (Subgroup III) (Tables 2, 3 and 4; Figs. 5, 6 and 7).

### Root segment influence

The effectiveness of smear layer removal followed a consistent spatial gradient: the cervical thirds displayed the most effective cleaning (lowest scores), the middle thirds showed intermediate results, and the apical thirds consistently exhibited the least effective removal (highest scores) among all study groups (Tables 2, 3 and 4; Figs. 2, 3, 4, 5, 6 and 7).

## Discussion

Among contemporary agitation systems, ultrasonic and laser-activated technologies are increasingly recognized for their capacity to enhance irrigant effectiveness and improve debridement of the dentinal surface [9, 12, 13, 16, 17, 21, 22]. However, their performance remains highly dependent on specific operational settings and the properties of the irrigation solution used [12, 13, 16, 17, 21, 22].

This investigation systematically compared four distinct irrigation protocols utilizing different chemical formulations: a sodium hypochlorite with EDTA combination, EDTA alone, sodium hypochlorite alone, and physiological saline as a negative control. The evaluation included CSN with side-vented needles alongside three activation techniques: (1) Er, Cr: YSGG laser (2780 nm) with a conical-end RFT, (2) a 976-nm diode laser with a plain tip, and (3) PUI. To our knowledge, this study represents the first comprehensive head-to-head comparison of 976-nm diode laser-activated irrigation, 2780-nm Er, Cr: YSGG laser-activated irrigation, and PUI for assessing smear layer removal efficacy. The evaluation was conducted across all root canal regions (coronal, middle, and apical thirds) using multiple irrigation protocols, enabling a complete assessment of each technique’s cleaning effectiveness under standardized conditions.

The distribution of the smear layer scores was assessed for normality using the Kolmogorov-Smirnov and Shapiro-Wilk tests. The results indicated a significant departure from normality across all experimental groups (p-values ranging from 0.003 to < 0.001), justifying the use of non-parametric statistical methods for all subsequent analyses. The reliability of the evaluative measurements was confirmed to be excellent. This was demonstrated by a Cohen’s kappa coefficient of 0.949, indicating near-perfect inter-examiner agreement, and intraclass correlation coefficients of 0.991, reflecting outstanding intra-examiner consistency. Furthermore, the high internal consistency of the dataset was supported by a Cronbach’s alpha value of 0.991. These robust reliability statistics affirm the reproducibility and consistency of the scoring methodology employed in this study.

The experimental results clearly demonstrated that Er, Cr: YSGG laser activation with RFT (1.2–2.7) surpassed all other irrigation methods tested (*p-*values ranging from 0.045 to < 0.001). This approach achieved significantly superior dentinal cleanliness compared to diode laser activation (1.6–3.6), PUI (2.3–4.4), and CSN irrigation (2.4–4.7), maintaining this advantage across all chemical solutions and root canal levels evaluated. These findings regarding the efficacy of laser-activated irrigation are consistent with previous literature, as multiple studies have confirmed its exceptional debridement capabilities [14, 32, 36, 40–43]. The superior performance of the Er, Cr: YSGG system appears to stem from its unique ability to generate vigorous fluid dynamics through two main mechanisms: intense transient cavitation phenomena and robust shock wave and acoustic streaming effects [16, 17, 22, 25, 27, 28]. These synergistic actions promote both deeper irrigant penetration into dentinal structures and more complete removal of organic and inorganic debris [14, 32, 36, 40–43].

These findings align with previous research demonstrating enhanced cleaning results through the photomechanical effects of Er, Cr: YSGG laser technology [14, 16, 17, 22, 25, 27–29, 36, 40–43], further supporting the clinical potential of laser-assisted endodontic irrigation [14, 32, 36–40]. Multiple studies, including those by George R. et al. (2008) [27], Sabari M. (2012) [40], Al-baker H. & Al-Huwaizi H. (2021) [30], Montero-Miralles P. et al. (2018) [27], Jameel C. & Zakaria N. (2020) [43], Rasheed S. & Jawad H. (2021) [36], and Abaza M. & Harhash T. (2024) [32], have found that Er, Cr: YSGG laser irradiation significantly improves the cleaning effectiveness of various chemical irrigants including peroxide, ethylenediaminetetraacetic acid-Cetavlon (EDTAC), water, normal saline, 2% chlorhexidine (CHX), 17% EDTA, and 3% and 5.25% NaOCl compared to non-activated irrigation. Notably, even alternative natural irrigants such as Salvadora persica and saline showed improved efficacy when activated by Er, Cr: YSGG laser, as reported by Abdelgawad L. et al. (2022a) [14]. Additionally, Al-Farawn M. et al. (2019) [44] and Varghese T. et al. (2025) [45] documented superior smear layer removal when 17% EDTA or 5.25% NaOCl was activated with Er, Cr: YSGG laser compared to diode lasers operating at 940 nm and 808 nm, respectively.

While not achieving the same efficacy as micro-pulsed Er, Cr: YSGG technology, 976-nm diode laser-activated irrigation still demonstrated significant advantages over CSN across all root canal regions and with all irrigants tested. The 976-nm diode laser improved smear layer removal compared to both PUI and non-activated controls (CSN with side-vented needle). This enhancement is primarily attributed to the photothermal effects of laser irradiation, which enhance irrigant agitation by improving solution kinetics and hydrodynamic activity while augmenting EDTA’s chelating action and NaOCl’s tissue-dissolving capacity [9, 16, 22, 46]. Additionally, diode laser irradiation may generate vapor bubbles within the solution and induce subsequent photomechanical waves that further improve the cleaning efficiency of root canal irrigants [46]. These results are consistent with previous studies confirming that diode laser-activated irrigation outperforms conventional techniques [20, 33, 46].

Furthermore, the application of 976-nm diode laser significantly enhanced the efficacy of all tested irrigants in smear layer elimination compared to PUI (*p-*values ranging from 0.045 to < 0.001) and CSN (*p-*values ranging from 0.021 to < 0.001) except with EDTA irrigant at middle thirds (*p*-values were 0.170 and 0.057 between diode laser and PUI and between diode laser and CSN respectively). This finding aligns with earlier scanning electron microscopy (SEM) studies that established diode laser-activated irrigation as more effective than ultrasonic activation for smear layer removal [36, 47, 48]. However, contrary to some studies reporting similar or superior smear layer removal capability with PUI compared to diode laser activation [43, 49, 50], these discrepancies may originate from variations in experimental protocols. Factors such as activation duration, application technique (including tip positioning), number of activation cycles, laser wavelength, beam parameters (power/pulse settings), and optical delivery systems may account for these observed differences [33, 43, 47–50].

PUI demonstrated insignificantly superior smear layer removal compared to CSN with a side-vented needle *(p-*values ranging from 1 to 0.075) except with saline irrigant at middle thirds where PUI showed significantly better score than that of CSN (*p* = 0.028). Previous research corroborates these findings, confirming PUI’s enhanced efficacy over syringe irrigation techniques [30, 37, 40]. This improved performance can be attributed to the mechanism of acoustic energy transfer from the oscillating ultrasonic file to the irrigant solution, which generates fluid cavitation and acoustic streaming. Consequently, PUI effectively dislodges debris and augments the irrigant’s ability to remove the smear layer. Furthermore, the reflux action produced during PUI facilitates the coronal displacement of debris [33, 47, 48].

While the Er, Cr: YSGG laser with a radial firing tip demonstrated statistically superior smear layer removal across all other irrigation methods tested, this performance must be contextualized with its practical drawbacks. The significant initial investment cost, technical training required, and unproper parameters can impact on dentin integrity are critical considerations for clinical adoption. In contrast, while the diode laser and PUI were less effective overall, their relative affordability and familiarity may make them a more viable option for many practices, particularly for coronal and middle third debridement.

While 976-nm diode laser has better water absorption than older diodes, it is still significantly lower than that of Er, Cr: YSGG, resulting in less potent cavitation and shockwave generation. PUI mechanism relies primarily on acoustic streaming, which is powerful but may be less effective than the explosive cavitation of laser systems for disrupting the smear layer, especially in the complex apical anatomy.

Comparative SEM analysis under identical irrigation protocols generally showed that the NaOCl + EDTA combination resulted in the greatest smear layer elimination, demonstrating the highest efficacy across all root segments, followed by 17% EDTA alone. Conversely, 5.25% NaOCl alone consistently underperformed compared to saline in all root regions when used with CNI, PUI, and diode laser-activated irrigation techniques. Particularly, across the groups, significant differences in irrigant efficacy were predominantly observed. For the Er, Cr: YSGG laser (group I), no significant differences were found between any irrigants in the coronal thirds *(p-*values ranging from 0.648 to 0.075). Significant differences were noted in the middle (*p* = 0.004) and apical thirds (*p* = 0.001), where NaOCl was significantly worse than the other solutions. In contrast, the performance of the 976-nm diode laser, PUI, and CSN (groups II-IV) was highly dependent on the irrigant. For these three techniques, NaOCl + EDTA was frequently significantly superior to NaOCl alone and saline. EDTA was also often significantly better than NaOCl (*p-*values ranging from 0.010 to < 0.001) and saline (*p-*values ranging from 0.042 to < 0.001). Furthermore, NaOCl was consistently significantly better than saline for the diode laser (group II) (*p-*values ranging from 0.028 to 0.027) and CSN (group IV) (*p-*values ranging from 0.042 to < 0.001). The only consistent non-significance was that NaOCl + EDTA and EDTA were often not significantly different from each other when used with PUI (Group III) (*p-*values ranging from 0.542 to 0.057) and CSN. (group V) (*p-*values ranging from 0.861 to 0.543).

The superior efficacy of the NaOCl-EDTA combination arises from their complementary chemical actions: EDTA dissolves the inorganic components, while NaOCl targets the organic matter within the radicular smear layer. Individually, each solution has limited effectiveness; EDTA alone only removes inorganic constituents, and NaOCl alone only addresses organic components. In contrast, normal saline lacks chemical reactivity with smear layer elements, making it incapable of significant smear layer elimination. These findings are supported by previous studies that have reported similar patterns of irrigant effectiveness [30, 37, 51, 52].

Notably, when Er, Cr: YSGG laser activation was applied, normal saline demonstrated superior smear layer removal efficacy compared to 5.25% NaOCl solution. This unexpected result indicates that saline interacts more effectively with Er, Cr: YSGG laser energy than NaOCl, resulting in significantly enhanced mechanical activation through cavitation, shockwave generation, and acoustic streaming that effectively dislodges smear layer from root canal dentin.

This finding suggests normal saline exhibits substantially greater interaction efficiency with Er, Cr: YSGG laser energy compared to 5.25% NaOCl. A 5.25% sodium hypochlorite solution is a higher concentration (by weight/volume) of solute than normal saline (0.9% NaCl). This means there are more solute ions per volume in the 5.25% NaOCl solution, which would slightly reduce the total number of water molecules in that same volume compared to a 0.9% saline solution. Thus, the 5.25% sodium hypochlorite solution has a lower concentration of free water molecules compared to saline. Consequently it would likely have slightly less overall Er, Cr: YSGG laser absorption than the same volume of normal saline, contradicting the initial premise. The primary effect of using the Er, Cr: YSGG laser in these solutions in clinical applications is related to the absorbed laser energy [23–26]. Higher water molecules in saline absorb more Er, Cr: YSGG laser energy and induce more mechanical effects [23–26]. In fact, the transferred light energy into mechanical one generates more vigorous mechanical activation effects, including intensive cavitation bubble formation, stronger shockwave propagation, and more pronounced acoustic streaming. These amplified physical phenomena collectively produce superior mechanical debridement, effectively dislodging and eliminating smear layer deposits from root canal dentin surfaces [23–26]. The differential response may be attributed to saline’s optimal affinity for Er, Cr: YSGG laser energy transfer as mentioned above, potential attenuation of laser effects by NaOCl’s chemical composition, or superior photon absorption characteristics in saline leading to more efficient photoacoustic conversion. This surprising finding corroborates previous research demonstrating similar differential effectiveness between saline and NaOCl when activated with Er, Cr: YSGG laser [14, 32].

The assessment of smear layer removal also revealed a distinct regional pattern, with cleaning efficacy decreasing progressively from coronal to apical regions. The highest smear layer scores (indicating poorest removal) were consistently observed in apical dentin (1.5–4.7), followed by intermediate values in middle thirds (1.2–4.2), while coronal sections demonstrated the most effective debridement (lowest scores) (1.2–3.8). This gradient suggests that the cleaning effectiveness of activated (laser and ultrasonic) and non-activated irrigation techniques diminishes significantly in apical regions. Several anatomical factors likely contribute to this phenomenon as the complex morphology of apical root anatomy, and the frequent presence of sclerotic dentin with reduced tubular permeability, a condition that typically progresses with age [29, 44]. These findings align with numerous previous investigations that have evaluated the performance of Er, Cr: YSGG and diode laser-activated, PUI, and CSN irrigation techniques across different root levels [14, 20, 32, 37, 41, 53, 54].

The findings of this in vitro study suggest several considerations for clinical practice. For clinicians seeking the highest level of debridement, the Er, Cr: YSGG laser with a radial-firing tip represents the most effective tool among those tested. Its ability to clean effectively even with saline could be advantageous in cases with open apices or perforations, where the use of cytotoxic irrigants like NaOCl is contraindicated and for patients with known hypersensitivity to sodium hypochlorite. Practices equipped with diode lasers can leverage them for enhanced irrigation. Using a 976-nm diode in pulsed mode as an adjunct to final irrigation can provide a significant improvement over conventional needle irrigation alone. The confirmed superiority of the NaOCl + EDTA combination reinforces its status as the gold-standard chemical regimen. Clinicians should ensure both solutions are used sequentially and are adequately activated to maximize their synergistic effect. The persistent difficulty in cleaning the apical third across all impedes the flow and penetration of sealer into the dentinal tubules and restrict the adaptation of the core material (e.g., gutta-percha) to the canal walls. The result is an imperfect seal that risks microleakage and potential long-term failure. Thus, it underscores the need for techniques that maximize mechanical agitation in this region, such as increased the used irrigant volume, the use of specialized laser tips or ultrasonic files designed to safely reach the apex without injuring the periapical tissues, and extended irrigation/activation time.

This in vitro investigation has several limitations that should be acknowledged. The study’s primary limitation is its exclusive focus on a physical outcome (smear layer removal) in an in vitro model. The absence of clinical variables, including microbial biofilms, and the lack of data on antibacterial efficacy and biocompatibility are notable gaps. The efficacy of an irrigation protocol is not solely defined by its cleaning ability but also by its capacity to eliminate the biofilm that causes apical periodontitis and its safety for periradicular tissues. Furthermore, the powerful photomechanical effects of Er, Cr: YSGG LAI, while excellent for debridement, raise questions about its impact on the dentin substrate. This study did not address whether these activation methods induce microcracks or alter the dentin matrix’s mechanical properties, which could potentially compromise the long-term strength of the root.

Also, the use of a homogenous sample of mandibular premolars with single, oval canals limits the direct generalizability of our results to teeth with more complex, multi-rooted anatomy. Moreover, sealing root apices with sticky wax to prevent irrigant extrusion simulates closed-end systems but may not fully replicate in vivo conditions, where periapical tissues and fluid dynamics influence irrigant behavior. The discrepancy in total fluid volume for the NaOCl + EDTA subgroup can influence the irrigants comparison results. Despite using a single, trained operator and a standardized protocol, the manual helical motion for laser and ultrasonic activation remains a potential source of variability compared to a fully automated system. Although only one half of the split root is the accepted and published standard, balancing practical constraints with scientific rigor, it is considered as a limit because of the examining both halves would provide the most comprehensive data. Regardless of SEM is the most established and commonly used method in the endodontic literature for smear layer removal, it does not allow macroscopic overview for the whole root canal surfaces. On the other hand, the absence of direct temperature measurement at the root surface remains a limitation, as SEM analysis cannot detect subtle, sub-ablation threshold temperature rises that could potentially affect the vital periodontal ligament in a clinical setting. The last limitation of this study is that it utilized a single set of parameters for each activation system. The potential for superior efficacy with different laser parameters, ultrasonic settings, laser tip sizes and designs or ultrasonic types remains unexplored.

Future studies should build upon these findings by incorporating complementary biological and materials science analyses. To assess antibacterial efficacy, established root canal biofilms should be used, adapting methodologies from studies that evaluate the antibacterial potency of activated agents, such as the model used by Kazi F. et al. (2024) [55]. For cytotoxicity evaluation, the eluates from dentin treated with these protocols should be tested on human periodontal ligament fibroblasts or stem cells of the apical papilla (SCAP), employing the gold-standard protocols established by Sequeira D. et al. (2021) [56] to provide indispensable safety data. To address the concern of dentin structural integrity, future work should employ microhardness testing or flexural strength measurements on dentin bars, similar to the material analyses performed by Amin F. et al. (2025) [57] and Mansoor A. et al. (2024) [58]. Subsequent clinical trials would then be necessary to validate these laboratory findings under actual clinical conditions.

Moreover, subsequent research should aim to enhance the clinical translatability of these in vitro findings by employing experimental models that more accurately recapitulate the complexities of the clinical environment. This includes using teeth with curved canals and complex multi-rooted anatomy to assess efficacy in challenging morphologies. Future studies should analyze both halves of each sample to provide comprehensive data and determine whether analyzing one half affects the results of smear layer removal. Advanced imaging techniques, such as micro-CT scanning, could be paired with SEM to provide a three-dimensional, quantitative analysis of debridement efficacy throughout the entire root canal system, overcoming SEM’s limitation of surface-specific evaluation. To optimize laser protocols, research should explore a wider range of laser parameters for both Er, Cr: YSGG and diode lasers, and employ automated, standardized tip movement systems to eliminate operator variability. Also, to maximize PUI technique, research should assess its efficacy when it is applied with other settings using different tips. The interaction of these advanced activation techniques with newer irrigant formulations, such as QMix, chitosan, or bioactive glass suspensions, warrants investigation. Following promising in vitro results, a logical progression would involve animal studies to evaluate the biological outcomes, including periapical tissue healing and biocompatibility, in response to these irrigation protocols. Ultimately, well-designed randomized controlled clinical trials are essential to validate the superior cleaning efficacy of laser-activated irrigation, particularly Er, Cr: YSGG with saline, and translate these laboratory findings into proven clinical benefits for long-term endodontic success.

Last but not least, while our SEM analysis revealed no signs of thermal damage—aligning with findings from other studies—future research incorporating direct thermocouple measurement is essential to fully characterize the thermal profile and conclusively establish the safety of these protocols under identical experimental conditions.

## Conclusion

Within the constraints of this in vitro investigation, the results demonstrated that Er, Cr: YSGG laser-activated irrigation surpassed both 976-nm diode laser activation and conventional needle irrigation in smear layer removal across all root canal levels when using the same irrigants. The 976-nm diode laser activation significantly improved smear layer removal compared to PUI and CSN. The synergistic combination of NaOCl and EDTA further enhanced debridement effectiveness, irrespective of the activation technique employed. Notably, saline solution activated by Er, Cr: YSGG laser unexpectedly removed more smear layer than NaOCl used alone, indicating a predominantly mechanical rather than chemical cleaning mechanism.

## Data Availability

No datasets were generated or analysed during the current study.
